# Reproductive and Oncologic Outcomes in Young Women with Stage IA and Grade 2 Endometrial Carcinoma Undergoing Fertility-Sparing Treatment: A Systematic Review

**DOI:** 10.3390/biom14030306

**Published:** 2024-03-05

**Authors:** Andrea Etrusco, Antonio Simone Laganà, Vito Chiantera, Mislav Mikuš, Hafiz Muhammad Arsalan, Antonio d’Amati, Amerigo Vitagliano, Ettore Cicinelli, Alessandro Favilli, Antonio D’Amato

**Affiliations:** 1 Unit of Obstetrics and Gynecology, “Paolo Giaccone” Hospital, 90127 Palermo, Italy; etruscoandrea@gmail.com (A.E.); antoniosimone.lagana@unipa.it (A.S.L.); 2 Department of Health Promotion, Mother and Child Care, Internal Medicine and Medical Specialties (PROMISE), University of Palermo, 90127 Palermo, Italy; vito.chiantera@unipa.it; 3 Unit of Gynecologic Oncology, National Cancer Institute—IRCCS—Fondazione “G. Pascale”, 80131 Naples, Italy; 4 Department of Obstetrics and Gynecology, Clinical Hospital Center Zagreb, 10 000 Zagreb, Croatia; m.mikus19@gmail.com; 5 Faculty of General Medicine, Altamimi Bachelor Clinical University, Bishkek 720000, Kyrgyzstan; dr.alsalan@altamimiedu.com; 6 Gynaecological Pathology Unit, Anatomical Pathology Division, Department of Woman and Child’s Health and Public Health Sciences, “Agostino Gemelli” Foundation IRCCS University Hospital, 00168 Rome, Italy; antonio.damati@uniba.it; 7 Unit of Human Anatomy and Histology, Department of Translational Biomedicine and Neuroscience (DiBraiN), University of Bari “Aldo Moro”, Piazza Giulio Cesare 11, 70124 Bari, Italy; 8 Unit of Obstetrics and Gynecology, Department of Interdisciplinary Medicine (DIM), University of Bari “Aldo Moro”, Policlinico of Bari, Piazza Giulio Cesare 11, 70124 Bari, Italy; amerigo.vitagliano@gmail.com (A.V.); ettorecicinelli@yahoo.it (E.C.); 9 Section of Obstetrics and Gynecology, Department of Medicine and Surgery, University of Perugia, 06123 Perugia, Italy; alessandro.favilli@unipg.it

**Keywords:** endometrial cancer, grade 2, fertility-sparing treatment, oncologic outcomes, reproductive outcomes, hormonal therapy, hysteroscopy

## Abstract

Background: Endometrial cancer (EC) is the most common gynecological malignancy in both Europe and the USA. Approximately 3–5% of cases occur in women of reproductive age. Fertility-sparing treatment (FST) options are available, but very limited evidence regarding grade 2 (G2) ECs exists in the current literature. This systematic review aimed to comprehensively evaluate reproductive and oncologic outcomes among young women diagnosed with stage IA or G2EC disease who underwent FST. Methods: A comprehensive search of the literature was carried out on the following databases: MEDLINE, EMBASE, Global Health, The Cochrane Library (Cochrane Database of Systematic Reviews, Cochrane Central Register of Controlled Trials, Cochrane Methodology Register), the Health Technology Assessment Database, and Web of Science. Only original studies that reported the oncologic and reproductive outcomes of patients with stage IA and G2EC tumors who underwent FST were considered eligible for inclusion in this systematic review (CRD42023484892). Studies describing only the FST for endometrial hyperplasia or G1 EC were excluded. Results: Twenty-two papers that met the abovementioned inclusion criteria were included in the present systematic review. Preliminary analysis suggested encouraging oncologic and reproductive outcomes after FST. Conclusions: The FST approach may represent a feasible and safe option for women of childbearing age diagnosed with G2EC. Despite these promising findings, cautious interpretation is warranted due to inherent limitations, including heterogeneity in study designs and potential biases. Further research with standardized methodologies and larger sample sizes is imperative for obtaining more robust conclusions.

## 1. Introduction

Endometrial carcinoma (EC) is the prevailing gynecological malignancy in both Europe and the USA and occurs at a rate ranging from 15 to 25 cases per 100,000 women in Western nations [1,2]. While predominantly affecting postmenopausal women, 25% of cases manifest in premenopausal individuals, and 3–5% occur in those under the age of 40 [3,4]. Moreover, the increasing incidence of obesity in women, in addition to leading to a higher incidence of classically associated diseases [5,6,7], has led to a surge in the incidence of EC. In fact, obesity is more strongly associated with the development of EC than any other type of cancer [8]. The primary therapeutic approach for EC traditionally involves total hysterectomy with bilateral salpingo-oophorectomy (THBSO). However, in specific cases, a shift toward fertility-preserving strategies is of paradigmatic importance. Currently, viable fertility-preserving options include the use of oral progestin agents, such as medroxyprogesterone acetate (MPA) or megestrol acetate (MA), as well as the application of the levonorgestrel-releasing intrauterine device (LNG-IUD), potentially in combination with hysteroscopic resection (HR) of the neoplasm [9]. Once complete remission is achieved and reproductive desires are met, patients should be strongly encouraged to undergo THBSO as definitive therapy [9]. This consideration is particularly pertinent for patients with FIGO stage IA low-grade (G1) tumors expressing a desire for future offspring [10,11].

Regarding the histological and molecular classifications of EC that have succeeded over the years, Bokhman proposed the first classification into Type I (endometrioid-type) and Type II (serous-type) in accordance with clinical, hormonal, and epidemiological observations [12,13]. Endometrial endometrioid carcinoma (EEC) is the predominant histological subtype of uterine body cancer across all age groups. These tumors are typically categorized into three histological groups characterized by variations in architectural and cytological atypia: grade 1 (G1), grade 2 (G2), and grade 3 (G3) [14]. In line with current practice standards, a FIGO grade is assigned to EECs based on the extent of glandular differentiation. Among tumors classified as G1, ≤5% had solid nonglandular, nonsquamous growth; among G2 tumors, this ranged from 6% to 50% and among G3 tumors, this surpassed the 50% threshold [15]. Statistical analyses utilizing classification and regression tree methods revealed that, following tumor stage, the most significant prognostic distinction in EEC lies in differentiating between high-grade (G3) and low-grade (G 1/2) tumors [16,17]. In this regard, several authors have emphasized the need to surpass the FIGO classification of cellular differentiation, replacing it with a binary grading system that may offer superior diagnostic efficacy and broader reproducibility [18,19,20]. Nevertheless, according to the latest guidelines of the European Society of Gynecological Oncology (ESGO)/European Society of Human Reproduction and Embryology (ESHRE)/European Society of Gynecological Endoscopy (ESGE), the three-grade-based grading system continues to be recommended [9]. The ProMisE classification, derived from the 2013 Cancer Genome Atlas (TCGA) Research Network [21] analysis, has potential for a significant role in determining patient prognosis and therapy, despite its current lack of clinical applicability [9,22].

Histologic grading has also been attributed great importance in the context of fertility-sparing treatments (FSTs). In fact, according to some authors, patients with a histologic grade higher than G1 have a lower complete response (CR) to conservative treatment and worse oncologic and reproductive outcomes [23]. Moreover, most of the studies currently available in the literature on FST for EC have focused on stage IA G1 endometrial tumors. In contrast, studies on the use of FST in stage IA G2 tumors are rare.

Therefore, the main purpose of this systematic review was to evaluate the oncologic and reproductive outcomes of women diagnosed with stage IA and grade 2 endometrial carcinoma (G2EC) who underwent FST.

## 2. Materials and Methods

### 2.1. Eligibility Criteria

Only original studies (retrospective or prospective) that reported oncologic and reproductive outcomes of patients with stage IA and G2EC disease who underwent FST were considered eligible for inclusion in this systematic review. Studies describing only the FST for endometrial hyperplasia (EH) or G1EC were excluded.

### 2.2. Information Sources

This study was carried out according to the Preferred Reporting Items for Systematic Reviews and Meta-Analyses (PRISMA) guidelines [24], available through the Enhancing the Quality and Transparency of Health Research (EQUATOR) network, and the Cochrane Handbook for Systematic Reviews [25]. The study was registered with the international prospective register of systematic reviews (PROSPERO) under the registration number CRD42023484892.

MEDLINE, EMBASE, Global Health, The Cochrane Library (Cochrane Database of Systematic Reviews, Cochrane Central Register of Controlled Trials, Cochrane Methodology Register), Health Technology Assessment Database, Web of Science, and Research Register (ClinicalTrial.gov) were searched for studies describing patients who underwent FST for stage IA or grade 2 EC.

### 2.3. Search Strategy

The following medical subject heading (MeSH) and key search terms were used: “Endometrial neoplasm” (MeSH Unique ID: D016889) AND “Grade 2” AND “Fertility sparing” (MeSH Unique ID: D059247) OR “Conservative treatment” (MeSH Unique ID: D000072700) AND “Hysteroscopy” (MeSH Unique ID: D015907) OR “Hysteroscopic surgery” (MeSH Unique ID: D015907) OR “Progestins” (MeSH Unique ID: D011372) OR “Levonorgestrel IUD” (MeSH Unique ID: D016912) OR “Megestrol Acetate” (MeSH Unique ID: D019290) OR “Medroxyprogesterone Acetate” (MeSH Unique ID: D017258) OR “Dydrogesterone” (MeSH Unique ID: D004394).

We selected papers written in English from the inception of each database until 31 December 2023.

### 2.4. Study Selection

Titles and/or abstracts of studies retrieved using the search strategy were screened independently by two review authors (A.E. and A.S.L.) to identify studies that met the inclusion criteria.

The full texts of these potentially eligible articles were retrieved and independently assessed for eligibility by two other review team members (M.M. and A.D.). A manual search of the references of the included studies was also conducted to prevent the omission of pertinent research.

Any disagreements between them over the eligibility of the articles were resolved through discussion with a third (external) collaborator. All of the authors approved the final selection.

### 2.5. Data Extraction

Two authors (A.V. and V.C.) independently extracted data from articles about study features, characteristics of the included populations, FSTs, complications, and results/outcomes using a prepiloted standard form to ensure consistency. One author (E.C.) reviewed the entire data extraction process.

### 2.6. Assessment of Risk of Bias

Two reviewers (H.M.A. and A.Da.) independently assessed the risk of bias of studies included in this systematic review using a modified version of the Newcastle–Ottawa Scale (NOS) [26]. The quality of the studies was evaluated in five different domains: “study design and sample representativeness”, “sampling technique”, “description of the fertility-sparing treatment”, “quality of the population description”, and “incomplete outcome data” (Table S1). Any disagreements between the reviewers were resolved by a third reviewer (A.E.).

### 2.7. Outcome Measures and Data Synthesis

The primary objective of this study was to evaluate the oncologic and reproductive outcomes of women with stage IA and grade 2 endometrial carcinoma who underwent FST. Quantitative analysis was not possible due to data heterogeneity (including differences in the type of FST). We provided a descriptive synthesis of the results in separate sections based on the type of FST that was employed: oral progestin therapy (oPT), LNG-IUD, HR, and combined treatment.

The body of evidence on the effectiveness of FST for IA G2EC was assessed by two authors (A.E., A.D.) using the Oxford Centre for Evidence-Based Medicine 2011 Levels of Evidence (OCEBM) [27].

## 3. Results

### 3.1. Study Selection

**Figure 1:**
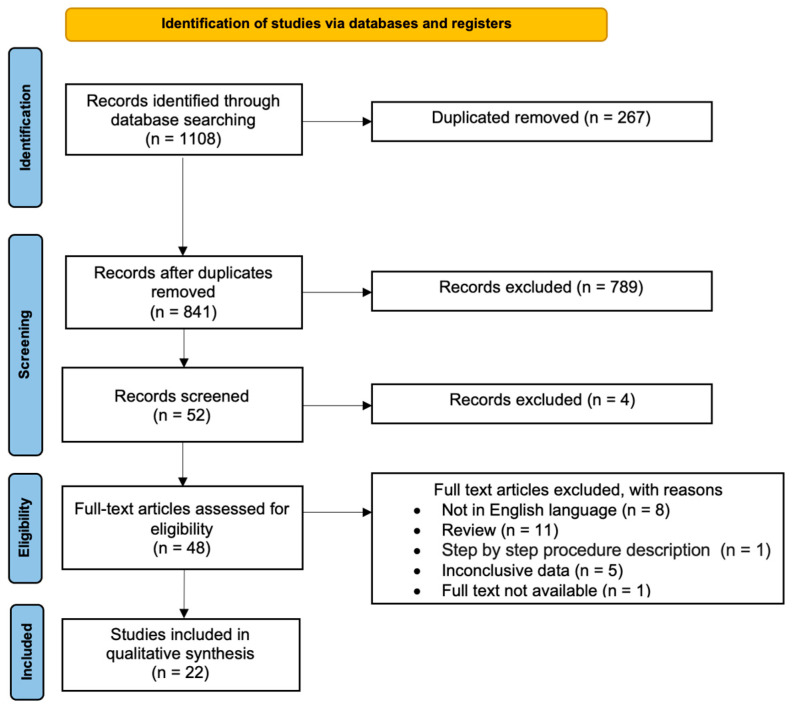
PRISMA flow diagram of the review.

The study selection process is displayed in Figure 1. After the evaluation of the full texts, 22 papers that met the abovementioned inclusion criteria [23,28,29,30,31,32,33,34,35,36,37,38,39,40,41,42,43,44,45,46,47,48] were included in the present systematic review.

### 3.2. Study Characteristics

The main characteristics of the included studies are summarized in Table 1. One study was a prospective study [38], thirteen were retrospective studies [23,28,30,31,32,33,34,35,39,41,42,43,47], four were case series [37,44,45,46], and four were case reports [29,36,40,48].

Of these, four studies were from Italy [30,38,43,44], five from Korea [23,33,36,39,42], three from China [32,46,47], three from the United States [29,40,41], two from Japan [34,35], two from Israel [31,48], one from France [37], one from Argentina [45], and one from Germany [28].

### 3.3. Risk of Bias of Included Studies

Of the twenty-two studies included, thirteen had a low risk of bias in three or more domains [23,28,29,30,32,33,35,36,38,40,43,46,47], and nine had a high risk of bias [31,34,37,39,41,42,44,45,48].

A detailed description of the risk of bias in each domain among the studies is reported in Table S2.

### 3.4. Synthesis of the Results

**Figure 2:**
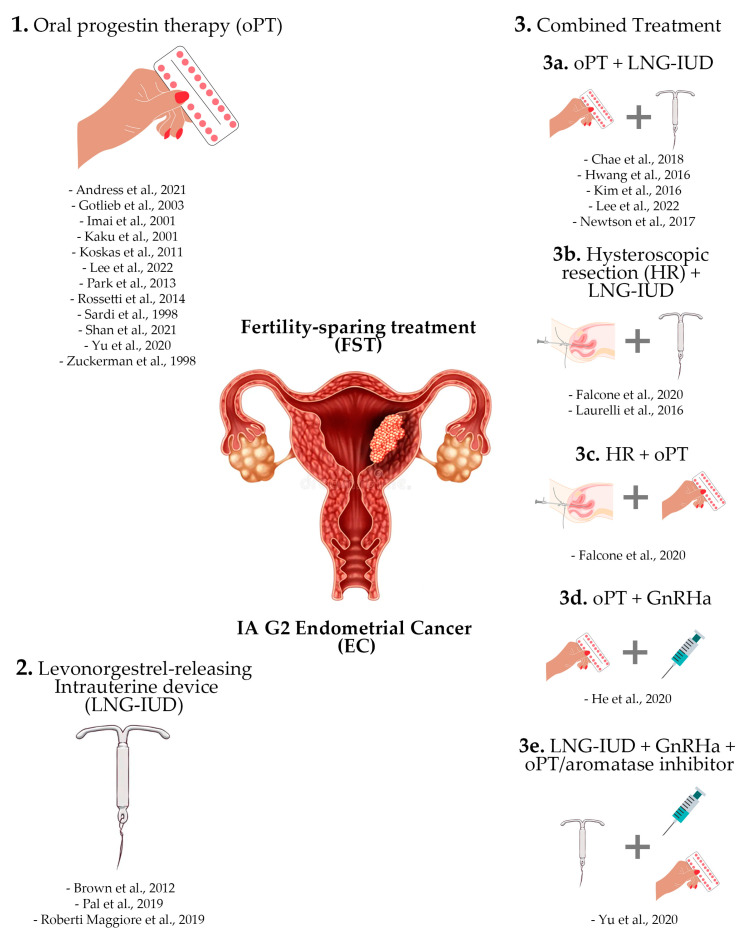
Summary of the various approaches used for the FST of IAG2 EC in the included studies. Twelve studies evaluated the use of oPT only for the FST of G2ECs [28,31,34,35,37,39,42,44,45,46,47,48]. Three studies analyzed oncologic and reproductive outcomes after the FST exclusively employing LNG-IUD [29,41,43]. Finally, Nine studies evaluated the use of a combined approach for determining the FST of G2ECs [23,30,32,33,36,38,39,40,47].

Among the included studies, twelve evaluated the use of oPT alone [28,31,34,35,37,39,42,44,45,46,47,48], three evaluated the use of LNG-IUD alone [29,41,43], and nine evaluated the use of combined treatment with oPT and LNG-IUD [23,32,33,36,39,40], HR and LNG-IUD [38], HR and LNG-IUD, oPT [30], or GnRH antagonists (GnRHa) and LNG-IUD [47]. The different approaches used for FTS of IAG2 EC in the included studies are summarized in Figure 2. Additional data on the main types of progestins and treatment regimens used in the included studies are given in Table 2.

Notably, in the studies by Lee et al. [39], Shan et al. [46], and Yu et al. [47], some patients received only oPT, while others underwent combined treatment. In the first two studies [39,46], the combinations included oPT and LNG-IUD, and in the third study [47], the combinations involved the use of oPT, GnRH antagonists (GnRHa), and LNG-IUD.

As previously mentioned, we discussed the results separately based on the type of FST approach used in the various included studies.

#### 3.4.1. Oral Progestin Therapy (oPT)

Twelve studies evaluated the use of oPT only for the FST of G2ECs [28,31,34,35,37,39,42,44,45,46,47,48]. Overall, the main objective was to evaluate the oncologic and reproductive outcomes after FST. Additional information regarding the included studies can be found in Table 2 and Table 3.

In chronological order, the first study was conducted by Sardi et al. in 1998 [45]. Four women with stage I EC, including one patient with G2EC, underwent FST with oPT. MPA 50 mg daily was administered. After 4 months of treatment, the patient did not show any response to oPT. No data were reported about subsequent surgical treatment or reproductive outcomes.

Yu et al. [47] combined different FST approaches for eight patients with G2EC. Six out of the eight patients were treated exclusively with oPT, MA 160 bid, or MPA 500 mg/day. All patients achieved CR except one who underwent hysterectomy. Two patients developed recurrence and were retreated with MA 160 mg bid for 4 or 6 months. One patient showed an AH, with a CR and NED after progestin retreatment. The other patient was diagnosed with G2-3 EC and showed SD after retreatment; therefore, she underwent radical surgery, which revealed IIIC1 G3 EC. Three patients tried to conceive immediately after achieving CR, and two patients achieved full-term delivery. Similar results were obtained by Zuckerman et al. [48], Gotlieb et al. [31], Rossetti et al. [44], and Shan et al. [46].

According to the series by Imai et al. [34], fifteen patients with EC, twelve with G1EC, two with G2EC and one with adenoacanthoma, were treated with oral MPA alone. One of the two patients with G2EC achieved a CR. Disaggregated data regarding reproductive outcomes were not available. Two out of fifteen patients conceived, resulting in three live births.

Kaku et al. [35] treated 30 patients with EC or AH with oPT. Twelve patients were diagnosed with EC, two of whom had G2EC. The oPT regimen involved 600 mg or 800 mg of MPA daily. The mean treatment duration for the two patients was 5 ± 1 month. One patient achieved a CR and had a full-term delivery after FST. The other patient showed stable disease (SD) at the end of the treatment and thus underwent total abdominal hysterectomy with right salpingo-oophorectomy. Both patients showed NED at the 19- and 22-month follow-ups.

In 2011, Koskas et al. [37] reported four cases of conservatively managed EC. Three women with G2EC and one with G3EC were included. No pregnancies occurred in the series.

Park et al. [42] retrospectively analyzed the oncologic and reproductive outcomes of 48 patients with IAEC of all grades subjected to FST with oPT. Of these, twenty-three (47.9%) were affected by G1EC, twenty-two (45.8%) by G2EC and three (6.3%) by G3EC. Disaggregated data for patients with G2EC were not available. ToPT regimen involved MA use for 14 patients (29.2%) and MPA for 34 patients (70.8%). Fourteen pregnancies occurred in the cohort; four miscarriages, one ectopic pregnancy, and nine live births occurred.

Andress et al. [28] evaluated the impact of oPTs on fourteen patients with stage I EC, of whom one had G2EC (7%), ten had G1EC (71%), and three (21%) had CAH.

Lee et al. [39] analyzed FST outcomes in 54 patients with EC, among whom 44 had G2EC (81.5%) treated with oPT alone or in combination with LNG-IUD. Disaggregated data for the 10 patients with G1EC were not available. Fifteen patients tried to conceive after CR, and seven pregnancies occurred—two of which resulted in abortions and five of which resulted in live births.

#### 3.4.2. Levonorgestrel-Releasing Intrauterine Device (LNG-IUD)

Three studies analyzed oncologic and reproductive outcomes after the FST exclusively employing LNG-IUD [29,41,43]. Further details about the included studies are presented in Table 4.

Brown et al. [29] reported the case of an 18-year-old patient diagnosed with a G2EC who was successfully treated by LNG-IUD placement.

Pal et al. [41] evaluated the use of LNG-IUD in 46 patients suffering from CAH and EC. Eight patients were diagnosed with G2EC (25%). Six months after LNG-IUD placement, among the patients with G2EC, three out of eight achieved a CR after treatment (37.5%), and three achieved a PR (37.5%). Two patients had SD (25%). Disaggregated data regarding reproductive outcomes were not available.

In the series by Roberti Maggiore et al. [43], forty-eight patients with ACH, G1EC or G2EC were treated with LNG-IUD, among whom four were diagnosed with G2EC (8.3%). Disaggregated data regarding the G2EC group were available. Three out of four patients achieved a CR (75%). No patients in the G2EC cohort who achieved a CR tried to conceive.

#### 3.4.3. Combined Treatment

Nine studies evaluated the use of a combined approach for determining the FST of G2ECs [23,30,32,33,36,38,39,40,47]. In seven out of nine studies, a combination of oPT and LNG-IUD was employed [23,32,33,36,39,40,47]. Two studies analyzed the outcomes after HR and LNG-IUD [30,38]. Additional data on the included articles can be found in Table 5.

Chae et al. [23] used a combination of oPT and LNG-IUD for the FST of 118 patients with stage I, grade 1–2 EC. Eleven patients were affected by G2EC. Disaggregated data for the G2 patients were not available. An analogous approach was employed by Hwang et al. [33] in five patients with G2EC. One pregnancy occurred in the series and culminated in a miscarriage.

Falcone et al. [30] reported their experience with the FST in 23 patients diagnosed with IAG2EC. A variety of different approaches were used. A CR was obtained in 17 patients (73.9%). Five pregnancies occurred; two were unsuccessful, and three were live births. He et al. evaluated the oncological and reproductive outcomes of 25 patients diagnosed with CAH, G1EC or G2EC. There were three patients (12%) with G2EC. Among the three patients, two were treated with oPT alone (250 mg/day), and the remaining patient received MPA 250 mg + GnRHa (3.75 mg/28 days). All three patients achieved a CR (100%). Unfortunately, disaggregated data regarding reproductive outcomes were not available.

Kim et al. [36] reported the case of a 13-year-old patient diagnosed with G2EC successfully treated with a combination of oPT (MA 160 mg + MPA 10 mg) and LNG-IUD. A similar result was obtained by Newtson et al. [40] on a 25-year-old patient.

Laurelli et al. [38] analyzed oncological and reproductive outcomes after FST in twenty-one patients with EC, of which one had G2EC. Patients were treated with HR and LNG-IUD placement in combination. After 6 months of treatment, endometrial sampling was performed, revealing progression; therefore, the patient underwent definitive surgery and had an IAG3 endometrioid EC at the final histological analysis.

Among the eight patients included in the study by Yu et al. [47], two were treated with a combined approach. No pregnancies occurred in the two cases.

Finally, it is noteworthy that in the series by Lee et al. [39], 31 patients (57.4%) also received LNG-IUD treatment, even though disaggregated data regarding oncologic and reproductive outcomes of G2EC patients were not available, as previously reported.

Quality of evidence: The evidence regarding the safety, effectiveness, and reliability of FST for G2EC was classified as evidence level 4.

## 4. Discussion

FST is a pivotal and evolving aspect of the management of EC, addressing the delicate balance between oncologic control and the preservation of reproductive potential. Women of childbearing age diagnosed with EC face a unique challenge, and it is imperative to explore the best therapeutic modalities that allow both cancer eradication and fertility preservation. As previously mentioned, approximately 5% of women diagnosed with EC are younger than 40 years old. Most patients exhibit early-stage tumors characterized by low-grade features and of the endometrioid subtype, usually confined to the endometrium [49,50]. The 5- and 10-year disease-specific survival rates for patients with stage I tumors were reported to be 99% and 98%, respectively [51]. Disease-specific survival rates are markedly greater in women of reproductive age than in older women, irrespective of additional prognostic factors, although the presence of comorbidities can negatively influence survival outcomes [52].

After tumor stage, the primary prognostic determinant in EECs is the distinction between high-grade (G3) and low-grade (G1/2) tumors [17]. In the clinical setting, the precise determination of FIGO grade is not always straightforward. The histopathological distinction of FIGO grades in EC patients presents numerous challenges. Many pathologists classify tight microacini with barely visible lumens as solid growth, despite the lack of specificity of FIGO grading rules [20]. The endorsement of a confluent microacinar pattern as “solid” growth for grading lacks evidence-based support. Although squamous differentiation is discounted as evidence of solid growth in the FIGO criteria, difficulties arise in grading tumors with solid growth resembling immature squamous epithelium or transitioning between nonkeratinizing squamous epithelium and spindle cell change [20]. In these cases, it is fair to assess the nuclear grade, starting with the glandular component and, if inconclusive, proceeding to the solid component [20]. Furthermore, a frequent and well-known challenge in grading involves determining the level and scope of nuclear atypia, which could elevate a tumor grade from one FIGO category to another [53]. In this regard, Zaino et al. suggested that a significant presence of severe nuclear atypia in the majority of cells (>50%) is necessary to reclassify a G1 or 2 EEC [14].

In 2013, the Cancer Genome Atlas (TCGA) Research Network conducted a comprehensive analysis of 373 EC samples [21], performing whole exome sequencing, transcriptome sequencing, genomic copy number analysis, protein array analysis, microsatellite stability testing, and methylation profiling. Based on their work, the molecular classification of EC was proposed, which delineates four distinct subtypes based on their genomic features: (i) DNA polymerase epsilon (POLE)/ultramutated (POLE), (ii) microsatellite-instable/hypermutated (MSI), (iii) copy-number-low/TP53-wild-type (CNL), and (iv) copy-number-high/TP53-mutant (CNH) subtypes. POLE ultramutated tumors, primarily endometrioid, have the most favorable prognosis and are associated with specific mutated genes, such as POLE, PTEN, PIK3R1, PIK3CA, FBXW7, KRAS, and TP53 [54]. The hypermutated type with MSI/mismatch repair deficiency (MMRd) group, linked to an intermediate prognosis, exhibited mutations in TP53, FBXW7, CTNNB1, ARID1A, PIK13CA, PIK3RI, PTEN, RPL22, PTEN, KRAS, ATR, CHK1, CDC5, Caspase 5, the BAX gene, and JAK1, which are prevalent in endometrioid EC [54,55,56]. The CNL subgroup, which has mutations in CTNNB1 and PTEN, is associated with an intermediate prognosis and endometrioid EC. Conversely, the CNH group, which has genomic instability and mutations in TP53, FBXW7, and PPP2R1A, is linked to an unfavorable prognosis and serous EC. To enhance the practical utility and applicability of the TCGA classification, more cost-effective alternatives to molecular prognostic markers (specifically, the immunohistochemical evaluation of MMR proteins and p53) were developed, and the Proactive Molecular Risk Classifier for Endometrial Cancer (ProMisE) was established by grouping four groups that mirror the TCGA prognostic categories: POLE-mutated (POLEmut), MMR-deficient (MMRd), p53-abnormal (p53abn), and no specific molecular profile (NSMP) [57]. The molecular classification could have meaningful implications both for FST management and prognostic value; for example, POLE-mutated carcinomas could respond better to conservative treatment; conversely, MSI/MMRd ECs are unlikely to respond to a similar approach or show a higher recurrence rate after initial regression, while in p53abn, an FST approach could be inappropriate [56,58,59].

More recently, Ran et al. [22] evaluated the oncologic outcomes of 106 patients with early-stage EC after ProMisE classification analysis. Twenty-three patients (21.7%) were classified as MMR-D, three (2.8%) as POLE-mutated, three (2.8%) as p53abn, and seventy-seven (72.6%) as p53wt. They found no significant difference in the CR rate (*p* = 0.152) or recurrence rate (*p* = 0.174) between the MMR-D and p53wt subtypes after FST, highlighting the possible absence of prognostic significance of the ProMisE classifier in EC patients who underwent FPT. At the outset, a better understanding of the clinical meaning of the molecular classification and larger prospective studies are needed to clarify the exact prognostic significance of the molecular classifier in these instances.

As widely discussed above, the current available options for FST in G2EC patients are oPT, LNG-IUD, HR, or a combination of the previous options. According to the 2023 ESGO/ESHRE/ESGE guidelines, the most effective FST involves a combined approach employing HR followed by the administration of oral progestins and/or LNG-IUD [9]. Notably, GnRHa should not be prioritized as a primary treatment option [9]. In the study by Falcone et al. [30] included in the present systematic review, 23 patients diagnosed with only IA G2EC were treated with a combined approach employing mainly HR, together with oPT and/or LNG-IUD. The CR rate was 73.9%, which is one of the highest reported rates in the current literature; however, the recurrence rate should be noted (41.2%).

MPA and MA are the safest and most effective oral progestin drugs for FST in patients with EC and are recommended at a dosage of 160–320 mg/day for MA and 400–600 mg/day for MPA [9]. Notably, MA and MPA constituted the predominant progestins utilized for FST across the studies analyzed in this systematic review. Among studies exclusively employing oPT for FST, MPA featured in five [34,35,44,45,48] and MA in two [31,46]. Additionally, Andress et al. [28] employed Dydrogesterone 10 mg, whereas Koskas et al. [37] utilized Norethisterone 20 mg, MA 160 mg, and Nomegestrol acetate 5 mg; furthermore, in two studies both MA or MPA were employed [42,52], while in one study MA was combined with GnRHa or LNG-IUD [47]. In studies employing a combined therapeutic approach for FST, MA and MPA remained the predominant progestin agents, often administered in conjunction with HR, GnRHa, and/or LNG-IUD. Dosages for MPA exhibited considerable variability, ranging from 50 mg to 800 mg, whereas MA was consistently administered at a dosage of 160 mg. Treatment durations also varied significantly, spanning from 3 to 11 months.

MA has demonstrated superior remission rates in comparison to MPA and other hormonal treatments [60]. This increased efficacy could be attributed to its increased bioavailability upon oral administration. The exact treatment duration remains undefined, although numerous studies indicate a median regression time of 4 to 6 months [23,32,43]. Factors such as obesity and insulin resistance, identified as risk factors, may necessitate an extended treatment duration. Consequently, a therapeutic timeframe of 6–12 months is proposed for achieving a CR [61]. In the absence of a response within this period, radical surgery is recommended [9,61]. The addition of metformin also appears to potentiate the effects of oPT therapy [46]. Future studies should clarify the actual role of metformin in treating oPTs in patients with EC and evaluate whether other molecules, such as inositols, may actually be useful in this setting [62].

LNG-IUD has shown a satisfactory remission rate and low recurrence rate, although its effectiveness has not been assessed in comparison with that of oral progestins [63]. Its use in combination with oPT, HR or GnRHa therapy showed increased cumulative efficacy in comparison to LNG-IUD alone [64]. The most commonly used technique for assessing HR is the three-step resection proposed by Mazzon et al. in 2005 [65], which consists of excision of the tumor lesion in step 1, excision of the endometrium surrounding the lesion (4–5 mm beyond) in step 2, and excision of the myometrium beneath the lesion (3–4 mm) in step 3. However, this technique requires proper training to be put into practice [66]. After the pathology report confirming low-grade EC without MI, a six-month treatment with or without LNG-IUD can be started [65]. To consider FST successful and advise attempting to conceive, two consecutive negative endometrial biopsies with a minimal interval of 3 months are needed [67]. If a PR is obtained after 6 months of therapy, treatment could be continued for an additional 3–6 months. If an SD is confirmed following a subsequent endometrial biopsy, patients should be counseled about whether to undergo hysterectomy [9,68].

Notably, the majority of high-quality evidence in the current literature pertains to the treatment of G1ECs. However, the evidence in the literature on G2EC is limited. On the one hand, this fact necessitates adequately informing the patient about therapeutic alternatives and discussing FST on a case-by-case basis [9]. On the other hand, these findings emphasize the need for further evidence regarding FST for G2EC.

In total, the oncological and reproductive outcomes of 157 women with stage Ia G2EC were evaluated. Ninety-one patients underwent the FST with oPT, thirteen patients underwent LNG-IUD, and fifty-three patients underwent combined treatments. After excluding two studies [39,42] that did not report disaggregated data, the effect of oPT could be evaluated in 25 patients. Nineteen (76%) of these patients were able to achieve CR over a treatment period ranging from 3 to 30 months. In contrast, PR or SD was recorded in six patients (24%). Of the patients in whom a CR was achieved, six (31.6%) experienced disease recurrence, with the time to recurrence ranging from 3 to 69 months. In addition to the two studies with disaggregated data, two other studies [34,45] did not report data on recurrence. Regarding reproductive outcomes, we were unable to reach robust conclusions on the actual need for ART and the rate of spontaneous pregnancy in patients who achieved CR and desired offspring, as many authors do not report these data. In this regard, various systematic reviews have underscored the significance of employing ART to facilitate conception in women who have undergone FST for EC or AH [63,69]. This approach aims to minimize the interval preceding definitive surgery, thereby mitigating the risk of relapse. Overall, there was a PR of 42.1%, with an LBR of 87.5% and an MR of 12.5. Notably, among the studies in which oPT was used only for FST, Park et al. [42] and Lee et al. [39] reported the highest PRs, 46.7% and 63.6%, respectively, and LBRs, 71.4% and 57.1%, respectively.

Considering that one study reported disaggregated data [41], it was only possible to evaluate the effect of FST treatment with LNG-IUD on five patients. Four of them (80%) achieved CR after a treatment period between 4 and 13 months. However, one patient (20%) had a recorded PR or SD (not specified). Among the four patients who achieved CR after 3 to 15 months, three (75%) experienced relapse. The effect of conservative LNG-IUD therapy on reproductive outcomes could not be verified, as none of these patients attempted conception.

Fifty-seven patients in total underwent FST treatment with combination therapy. However, due to the presence of a study with disaggregated data [23], the oncologic and reproductive outcomes of only 42 patients were evaluated. CR was achieved in 33 patients (78.6%) after conservative combination therapy, ranging from 3 to 77 months. However, nine patients (21.4%) reported a PR or SD despite treatment. Finally, 11 patients (33.33%), after achieving CR, experienced recurrence from 4 to 41 months after discontinuation of FST. Given the results of an additional study with disaggregated data [32] and the low number of patients who desired pregnancy, the effect of combined FST on reproductive outcomes could be assessed in only 15 patients. There were four (50%) spontaneous pregnancies and four (50%) spontaneous pregnancies associated with ART after FST. Overall, there was a PR of 53.3%, an LBR of 62.5%, and an MR of 37.5%. In particular, among the authors who applied a combined approach, Chae et al. [23] reported a PR of 61.2% and an LBR of 42.9%, while in He et al. [32], the PR was 57.1%, and the LBR was 42.9%. In the study by Falcone et al. [30], in which only patients affected by G2EC were included, the percentage of patients with a PR was 50%, and the LBR was 30%. The mean age of the individuals in the cohort was 34.6 ± 4.73 years.

In addition, with regard to combined treatments, it would be appropriate for future studies to establish the role of new technologies in the conservative treatment of ECs [70,71].

These results are consistent with those of a meta-analysis by Herrera Cappelletti et al. [72], in which the LBR in women affected by early-stage EC under the age of 35 was 30.7%, while in women over the age of 40, it was 23.0%. With regard to the relationship between reproductive outcomes and the various FST approaches, the FST using oPT exhibited greater PRs and LBRs than did the LNG-IUD alone.

Moreover, a meta-analysis of 28 studies (1038 patients) revealed 34% PR and 20% LBR with oral progestin, compared to 18% and 14%, respectively, with only the LNG-IUD [73]. Combined treatment (HR followed by hormonal therapy) achieved higher LBRs than oral progestogen alone, with a meta-analysis reporting 53% in the hysteroscopy group versus 33% in the progestin-only group [74]. For any woman seeking pregnancy, age appears to be the most important factor influencing reproductive outcome. However, additional studies are needed to precisely assess the PR and LBR in G2EC-affected patients only.

Our systematic review, despite all of the limitations of the absence of disaggregated data for many studies and the absence of large patient cohorts, showed that the FST with LNG-IUD was the best in terms of CR and recurrence rate. In terms of reproductive outcomes, only the FST with oPT and the combined FST could be compared. Although the FST with only oPT was associated with a lower PR (42.1% vs. 53.3%), it was better than the combined treatment in terms of LBR (87.5% vs. 62.5%) and MR (12.5% vs. 375%).

Hence, although the 2023 ESGO/ESHRE/ESGE guidelines affirm that the combined approach stands out as the superior FST, demonstrating superior outcomes in terms of both CR and LBRs when compared to alternative treatment modalities [9], it is important to note that they refer to EC IAG1. Our systematic review, conversely, with all of the inherent limitations, demonstrates that for IA G2EC, the LNG-IUD would lead to the best oncological outcomes, while oPT alone would show the best reproductive outcomes.

A final remark should be made about counseling, which is of paramount importance for these patients. Patients with stage I G2EC must understand that the conservative treatment offered is outside any available guidelines or recommendations. Oncologic risk is not well defined, and stage I diagnosis is based solely and exclusively on the analysis of nuclear magnetic resonance images and not on a definitive histologic piece. In addition, although endometrial mapping may provide a valid method for establishing tumor spread within the uterine cavity and histologic grading, it is possible that some areas may not be sampled and that some areas among the nonsampled areas may have even worse histologic grading. In addition, patients should be informed of the risk of failing to achieve spontaneous pregnancy and that ART may be necessary [63,69]. In this sense, in the specific case of G2ECs, G2 syndrome appears to be related to worse reproductive outcomes as well as worse oncologic outcomes. Finally, once their desire for offspring is fulfilled, patients should be strongly motivated to undergo definitive surgical treatment to reduce oncological risks. On this last point, however, there is much debate. How much hysterectomy is necessary in patients who achieve complete remission after FST treatment and who have achieved one or more pregnancies? How much can the progesterone load during pregnancy be a method of sending the neoplasm into complete remission? Future studies should focus on these questions to ensure that the best care is provided, oncological and reproductive, for young patients forced to face such a crucial challenge as cancer.

### Strength and Limitations

To the best of our knowledge, this is the most comprehensive and up-to-date systematic review investigating the efficacy, safety, and feasibility of FST for G2EC. A multitude of conservative treatment options and combinations thereof were comprehensively analyzed. Oncologic and reproductive outcomes were also evaluated for each treatment or combination of treatments. However, although it was possible to determine oncologic and reproductive outcomes based on the technique used, the presence of disaggregated and often incomplete data and follow-ups that were not very long did not allow solid conclusions to be reached.

The primary constraint In the present systematic review lies in the variability of study designs, patient populations, and treatment protocols across the reviewed literature. Heterogeneity in FSTs, follow-up durations, and outcome measures introduces challenges in directly comparing results and drawing universal conclusions. Another significant limitation consists of the lack of disaggregated data regarding patients affected only by G2EC in several studies in which reproductive and oncological outcomes were analyzed in a heterogeneous cohort of patients affected by “early-stage EC”. Additionally, the absence of high-quality randomized controlled trials (RCTs) and the great predominance of retrospective studies may introduce selection biases and confounding factors that influence the interpretation of both reproductive and oncologic outcomes. The potential for publication bias may impact the robustness of the evidence base. Furthermore, the relatively small sample sizes in some studies might limit the generalizability of the findings.

## 5. Conclusions

In conclusion, this systematic review sheds light on the reproductive and oncologic outcomes of young women diagnosed with stage IA and grade 2 EC who underwent FST. An analysis of the available literature revealed promising results, emphasizing the feasibility of fertility-sparing approaches in this specific patient population.

The analysis of the available literature revealed promising results, underscoring the feasibility of fertility-sparing approaches in women affected by stage IA G2EC. The evidence suggests favorable reproductive outcomes, with higher PRs and LBRs observed after FST with oPT than after the combination treatment. Additionally, the oncologic outcomes demonstrated encouraging results, supporting the safety of these approaches in managing early-stage G2ECs.

However, further research and larger-scale studies, possibly with randomized or prospective designs and better patient selection criteria, are warranted to refine treatment strategies and provide more robust evidence to guide clinical decision-making in this unique cohort of women.

On the other hand, thorough discussions of reproductive-age women diagnosed with G2EC about their individual case, prognosis, and fertility-sparing options are needed. The decision to pursue FST should be made collaboratively, taking into account the patient’s medical condition, personal preferences, and long-term reproductive goals. FST results for G2EC-affected patients should be discussed case by case, as highlighted by the latest guidelines.

## Figures and Tables

**Table 1:** Characteristics of the included studies.

Author	Year	Type	Main Outcome	Country	Patient (n)	G2EC (n)	Age (Mean or Median)
Andress et al. [28]	2021	Retrospective	Oncologic and reproductive outcome of FST with oPT in early-stage G1EC, G2EC, or CAH.	Germany	14	1	34.2
Brown et al. [29]	2012	Case report	Report of an 18-year-old woman diagnosed with a G2EC treated with LNG-IUD.	United States	1	1	18
Chae et al. [23]	2018	Retrospective	Oncologic and reproductive outcomes after combined treatment for endometrioid EC.	Korea	118	11	37 (28–45)
Falcone et al. [30]	2020	Retrospective	Oncologic and reproductive outcomes of a combined FST for IA G2EC patients.	Italy	23	23	34.6
Gotlieb et al. [31]	2003	Retrospective	Oncologic and reproductive outcomes of oPT for patients with EC of all grades.	Israel	13	1	2 5
He et al. [32]	2020	Retrospective	Safety and efficacy of fertility- preserving retreatment in patients with AEH and EC after recurrence following initial FST.	China	110	3	32.84
Hwang et al. [33]	2016	Retrospective	Oncologic and reproductive outcomes of combined oPTLNG-IUD treatment in young women with IA G2EC.	Korea	5	5	30.4
Imai et al. [34]	2001	Retrospective	Effectiveness of oPT for FST in patients with EC.	Japan	15	2	n.d.
Kaku et al. [35]	2001	Retrospective	Oncologic and reproductive outcome after oPT for FST in patients with EC.	Japan	39	2	29.3 (21–42)
Kim et al. [36]	2016	Case report	Report of a case of EC occurred in a 13-year-old girl treated with hormonal therapy.	Korea	1	1	13
Koskas et al. [37]	2011	Case series	Report of four cases of G2-3 ECs managed conservatively to preserve fertility.	France	4	3	37.5
Laurelli et al. [38]	2016	Prospective	Oncologic and reproductive outcomes in EC in young patients conservatively treated by combined HR and LNG-IUD.	Italy	21	1	35.9
Lee et al. [39]	2022	Retrospective	Oncologic and pregnancy outcomes of FST using oPT with/without LNG-IUD in patients with stage I G2 endometrioid EC without MI or G1–2 with superficial MI.	Korea	54	46	34 (18–44)
Newtson et al. [40]	2017	Case report	Report of a case of successful FST in a young woman with G2EC refractory to single agent progestin treatment.	United States	1	1	25
Pal et al. [41]	2019	Retrospective	To assess efficacy of the LNG-IUD for treatment of CAH or low-grade EC.	United States	46	8	47.1 (18.5–85.2)
Park et al. [42]	2013	Retrospective	Oncologic and reproductive outcomes after oPT of women with EC IA, G1 with superficial MI or IA, G2–3 with or without superficial MI.	Korea	48	22	30 (23–40)
Roberti Maggiore et al. [43]	2019	Retrospective	Oncologic and reproductive outcomes of LNG-IUS treatment in patients affected by ACH/EC.	Italy	48	4	34.5
Rossetti et al. [44]	2014	Case series	Report of five cases of successful FST of early-stage EC.	Italy	5	2	30 (27–31)
Sardi et al. [45]	1998	Case series	Report and reproductive outcomes of 4 cases of EC treated with FST.	Argentina	4	1	32
Shan et al. [46]	2021	Case series	Clinical outcomes of oPT alone or plus metformin in young women with IA G2EC.	China	4	4	34.25
Yu et al. [47]	2020	Retrospective	Efficacy of FST for women with IA G2EC.	China	8	8	26 (22–35)
Zuckerman et al. [48]	1998	Case report	Healthy twin pregnancy after FST with oPT for G2EC.	Israel	1	1	26

FST—fertility-sparing treatment; oPT—oral progestin therapy; EC—endometrial cancer; G1EC—grade 1 endometrial cancer; G2EC—grade 2 endometrial cancer; CAH—complex atypical hyperplasia; LNG-IUD—levonorgestrel-releasing intrauterine device; AEH—atypical endometrial hyperplasia; HR—hysteroscopic resection; MI—myometrial invasion.

**Table 2:** Additional data on the main types of progestins and treatment regimens used in the included studies.

Author	Patients with G2EC (n)	Progestin Employed (% of Patients Treated)	Posology	Dosage (mg, % of Patients Treated)	Treatment Duration (Months, Mean or Median)
Andress et al. [28]	1	Dydrogesterone (100)	n.d.	10 (100)	3
Gotlieb et al. [31]	1	MA (100)	Once daily for two weeks a month	200 (100)	3
Imai et al. [34]	2	MPA (100)	n.d.	600	6 ± 3
Kaku et al. [35]	2	MPA (100)	n.d.	Case 1: 600 Case 2: 800	5 ± 1
Koskas et al. [37]	3	Case 1: Norethisterone (33.3) Case 2: MA (33.3) Case 3: Nomegestrol acetate (33.3)	Once daily	Case 1: 20 mg (33.3) Case 2: 160 mg (33.3) Case 3: 5 mg (33.3)	4.7 ± 1.2
Lee et al. [39]	44	MA (18.5) MPA (81.5)	Once or twice daily	MA 160 (11.2) 320 (3.7) 800 (1.8) 40 (1.8) MPA 500 (55.6) 1000 (25.9)	11 (3–30)
Park et al. [42]	22	MA (29.2) MPA (70.8)	Once daily	MA Median 160, range 40–240 MPA Median 500, range 80–1000	10 (3–20)
Rossetti et al. [44]	2	MPA (100)	Once daily	160 (100)	6
Sardi et al. [45]	1	MPA (100)		50 (100)	4
Shan et al. [46]	4	MA (75) MA + LNG-IUD (25)	Once daily	160 (100)	6.6 ± 3.4
Yu et al. [47]	8	MPA (37.5) MA (37.5) MA + LNG-IUD + GnRHa (12.5) GnRHa + LNG-IUD + aromatase inhibitor (12.5)	MA/MPA Twice daily MPA Once daily GnRHa Every 4 weeks	MPA 500 (37.5) MA 160 (50)	4.7 ± 2.2
Zuckerman et al. [48]	1	MPA	n.d.	n.d.	2
Chae et al. [23]	11	MPA + LNG-IUD (100)	Once daily	500–1000 (100)	12.2 (3–49)
Falcone et al. [30]	23	MA (4.3) MA + HR (21.7) MA + LNG-IUD (4.3)	Once daily	160 mg (100)	17.3 ± 16.9
He et al. [32]	3	MPA (66.7) MPA + GnRHa (33.3)	Once daily	250 (100)	5 (3–18)
Hwang et al. [33]	5	MPA + LNG-IUD (100)	Once daily	500 (100)	11 ± 6.2
Kim et al. [36]	1	MA + MPA + LNG-IUD (100)	Once daily	MA 160 (100) MPA 10 (100)	MA 3 MPA 5
Newtson et al. [40]	1	MA + LNG-IUD	Twice daily	80 mg (100)	10

MA—megestrol acetate; MPA—medroxyprogesterone acetate; LNG-IUD—levonorgestrel-releasing intra-uterine device; GnRHa—Gonadotropin Releasing Hormone antagonist.

**Table 3:** (**a**) Oncological outcomes of studies reporting the use of oPT for FST of IA G2 ECs. (**b**) Reproductive outcomes of studies reporting the use of oPT for the FST of IA G2 ECs.

**(a)**												
	**Andress et al.** [28]	**Gotlieb et al.** [31]	**Imai et al.** [34]	**Kaku et al.** [35]	**Koskas et al.** [37]	**Lee et al.** [39] *	**Park et al.** [42] *	**Rossetti et al.** [44]	**Sardi et al.** [45]	**Shan et al.** [46]	**Yu et al.** [47]	**Zuckerman et al.** [48]
**Patients, n**	14	13	15	30	4	54	48	5	4	4	8	1
**G2EC, n (%)**	1 (7.1)	1 (7.7)	2 (13.3)	2 (6.6)	3 (75)	44 (81.5)	22 (45.8)	2 (40)	1 (25)	4 (100)	8 (100)	1 (100)
**Age (years, mean or median)**	35.9	25	n.d.	31.5 ± 1.5	36 ± 3.7	34 (18–44)	30 (23–40)	n.d.	32	34.25 ± 3.77	27.6 ± 4.4	26
**BMI, kg/m^2^ (mean)**	42.8	n.d.	n.d.	n.d.	n.d.	24 (16–40)	23.6 (18.6–38.2)	n.d.	n.d.	26.07 ± 7.13	30 ± 4.1	n.d.
**oPT regimen (% of patients treated)**	Dydrogesteron 10 mg (100)	MA 200 mg daily two weeks a month (100)	MPA 600 mg (100)	MPA 600–800 mg (100)	Case 1: Norethisterone 20 mg (33.3) Case 2: MA 160 mg (33.3) Case 3: Nomegestrol acetate 5 mg (33.3)	MA 160 mg (11.2) MA 320 mg (3.7) MA 800 mg (1.8) MA 40 mg (1.8) MPA 500 mg (55.6) MPA 1000 mg (25.9)	MA 160 mg (40–240 mg) (14/48, 29.2); MPA 500 mg (80–1000 mg) (34/48, 70.2)	MPA 160 mg (100)	MPA 50 mg (100)	MA 160 mg + Metformin (50) MA 160 mg (25) MA 160 mg + Metformin + LNG-IUD (25)	MPA 500 mg/day (37.5) MA 160 mg (37.5) MA 160 + GnRHa + LNG IUD (12.5) GnRHa + LNG-IUD + aromatase inhibitor (12.5)	MPA (n.d. about dosage, 100)
**Treatment duration (months, mean or median)**	3	3	6 ± 3	5 ± 1	4.7 ± 1.2	11 (3–30)	10 (3–20)	6	4	6.6 ± 3.4	4.7 ± 2.2	2
**CR, n (%)**	0 (0)	1 (100)	1 (50)	1 (50)	3 (100)	33 (75)	36 (74.1)	2 (100)	0 (0)	3 (75)	7 (87.5)	1 (100)
**Time to** **CR (months, mean or median)**	0	3	29 (n.d. about range)	12	4.7 ± 1.2	10 (3–24)	17 (9–51)	6	nd	6.6 ± 3.4	4.7 ± 2.2	n.d.
**PR or SD, n (%)**	1 (100)	0 (0)	1 (50)	1 (50)	0 (0)	11 (25)	12 (25.8)	0 (0)	1 (100)	1 (25)	1 (12.5)	0 (0)
**No. of recurrence, n (%)**	0 (0)	1 (100)	n.d.	0 (0)	2 (66.6)	15 (38.5)	16 (33.3)	0 (0)	nd	0 (0)	3 (37.5)	0 (0)
**Time to recurrence (months)**	-	40	n.d.	0	Case 1: 6 Case 2: 36	23 (3–101)	Group 1: 19 (8–20) Group 2: 18 (7–69) Group 3: 34 (14–48)	-	-	-	25.7 ± 7.9	0
**Recurrence diagnosis (%)**	-	n.d.	n.d.	0	G1EC (100)	G1EC (80) G2EC (20)	CAH (6.3) G1EC (69) G2EC (6.3) G3EC (12.5)	-	-	-	AH (66.6) G2-3EC (33.3)	0
**oPT retreatment, n (%)**	-	1 (100)	n.d.	-	-	9 (60)	13 (81.3)	-	-	-	2 (66.7)	-
**Response to oPT retreatment, n, % (months)**	-	1, 100 (37)	n.d.	n.d.	n.d.	n.d.	11, 84.6 (n.d.)	n.d.	n.d.	n.d.	2, 66.6 (4.3 ± 1.2)	-
**Hysterectomy, n (%)**	1 (100)	1 (100%)	n.d.	1(50)	1 (50)	11 (20.4)	5 (38.5)	1 (50)	n.d.	1 (25)	3 (37.5)	0 (0)
**Follow-up** **(n,%)**	NED	NED	n.d.	NED (100)	Case 1: G1EC Case 2: NED Case 3: NED	NED (45, 83.3) AWD (14.8) Death (1.9)	NED (100)	NED	n.d.	NED (100)	NED (100)	n.d.
**Follow-up time (months)**	5	94	n.d.	20.5 ± 1.5	30 ± 22.4	44 (1–132)	Group 1: 8 (7–136) Group 2: 49 (22–95) Group 3: 76 (36–99	n.d.	n.d.	29.8 ± 16.2	36.2 ± 16.1	-
**(b)**												
	**Andress et al.** [28]	**Gotlieb et al.** [31]	**Imai et al.** [34] *	**Kaku et al.** [35]	**Koskas et al.** [37]	**Lee et al.** [39] *	**Park et al.** [42] *	**Rossetti et al.** [44]	**Sardi et al.** [45]	**Shan et al.** [46]	**Yu et al.** [47]	**Zuckerman et al.** [48]
**Patients, n**	14	1	15	2	4	54	48	2	1	4	8	1
**G2EC, n (%)**	1 (7.1)	1 (7.7)	2 (13.3)	2 (6.6)	3 (75)	44 (81.5)	22 (45.8)	2 (40)	1 (25)	4 (100)	8 (100)	1 (100)
**Nulliparous, n (%)**	11 (78.6)	1 (100)	n.d.	100	4 (100)	54 (100)	46 (96)	n.d.	n.d.	n.d.	n.d.	0 (0)
**Primiparous, n (%)**	3 (21.4)	0	n.d.	0	0	0	2 (4)	n.d.	n.d.	n.d.	n.d.	1 (100)
**History of infertility, n (%)**	11 (78.6)	n.d.	n.d.	n.d.	n.d.	11 (20.4)	17 (35.4)	2 (100)	n.d.	2 (50)	3 (37.5)	0 (0)
**Attempted to conceive, n (%)**	n.d.	1 (100)	n.d.	1 (50)	1 (25)	15 (38.5)	22 (46)	2 (100)	n.d.	3 (75)	3 (37.5)	1 (100)
**Time to conception attempt after CR (months)**	n.d.	n.d.	n.d.	n.d.	6	n.d.	1 (1–25)	n.d.	n.d.	n.d.	n.d.	n.d.
**ART, n (%)**	n.d.	n.d.	n.d.	n.d.	0 (0)	n.d.	12 (54.5)	2 (100)	n.d.	2 (66.6)	n.d.	0 (0)
**Pregnancies, n**	1	1	2	1	1	7	14	2	n.d.	1	2	1
**Miscarriages, n (%)**	1 (100)	0 (0)	0 (0)	0 (0)	0 (0)	2 (28.6)	4 (29)	0 (0)	n.d.	0 (0)	0 (0)	0 (0)
**Ectopic pregnancies, n (%)**	0 (0)	0 (0)	0 (0)	0 (0)	0 (0)	0 (0)	1 (7)	0 (0)	n.d.	0 (0)	0 (0)	0 (0)
**Twin pregnancy, n (%)**	0 (0)	0 (0)	1 (50)	0 (0)	1 (100)	0 (0)	1 (7)	0 (0)	n.d.	0 (0)	0 (0)	1 (100)
**Preterm delivery, n (%)**	0 (0)	0 (0)	n.d.	0 (0)	n.d.	0 (0)	1 (7)	0 (0)	n.d.	0 (0)	0 (0)	0 (0)
**Full-term delivery (%)**	0 (0)	1 (100)	n.d.	1 (100)	n.d.	5 (100)	8 (57)	2 (100)	n.d.	1 (100)	2 (100)	1 (100)
**Live births, n (%)**	0 (0)	1 (100)	3 (100)	1 (100)	2 (100)	5 (100)	9 (64.3)	2 (100)	n.d.	1 (100)	2 (100)	2 (100)
**PR (%)**	7.1	100	13.3	50	25	46.7	63.6	100	n.d.	33.3	25	100
**MR (%)**	100	0	0	0	0	28.6	28.6	0	n.d.	0	0	0
**LBR (%)**	0	100	100	100	100	71.4	57.1	100	n.d.	33.3	100	100

(**a**) EC—endometrial cancer; AH—atypical hyperplasia; CAH—complex atypical hyperplasia; G1EC—grade 1 endometrial cancer; G2EC—grade 2 endometrial cancer; G3EC—grade 3 endometrial cancer; BMI—body mass index; oPT—oral progestin therapy; MA—megestrol acetate; MPA—medroxyprogesterone acetate; GnRHa—gonadotropin releasing hormone antagonist; LNG-IUD—levonorgestrel-releasing intrauterine device; CR—complete response; PR—partial response; SD—stable disease; NED—nonevidence of disease; AWD—live with disease. * For these studies, disaggregated data were not available. (**b**) CR—complete response; ART—assisted reproductive technology; PR—pregnancy rate; MR— miscarriage rate; LBR—live birth rate; CLBR—cumulative live birth rate. * For these studies, disaggregated data were not available.

**Table 4:** (**a**) Oncological outcomes of studies reporting the use of LNG-IUD for the FST of IA G2 ECs. (**b**) Reproductive outcomes of studies reporting the use of the LNG-IUD for the FST of IA G2 ECs.

**(a)**			
	**Brown et al.** [29]	**Pal et al.** [41] *	**Roberti Maggiore et al.** [43]
**Patients, n**	1	46	48
**G2EC, n (%)**	1 (100)	8 (17.4)	4 (8.3)
**Age (years, mean or median)**	18	47.1 (18.5–85.2)	34.5 ± 3.3
**BMI, kg/m^2^ (mean)**	47.7	45 (19–74)	31.3 ± 14.5
**IUD**	LNG	LNG	LNG
**Treatment duration (months, mean or median)**	13	6	4
**CR, n (%)**	1 (100)	17 (37.5)	3 (75)
**Time to** **CR (months, mean or median)**	13	6	4.0 ± 0
**PR or SD, n (%)**	0 (0)	29 (62.5)	1 (25)
**No. of recurrence (%)**	0	n.d.	3 (75)
**Time to recurrence (months)**	3	n.d.	14.3 ± 1.5
**Recurrence diagnosis (%)**	-	n.d.	n.d.
**Retreatment (%)**	-	n.d.	n.d.
**Response to retreatment, n, % (months)**	-	n.d.	n.d.
**Hysterectomy, n (%)**	0 (0)	n.d.	n.d.
**Follow-up (n, %)**	NED	n.d.	n.d.
**Follow-up time (months)**	13	n.d.	115.5 ± 2.6
**(b)**			
	**Brown et al.** [29]	**Pal et al.** [41] *	**Roberti Maggiore et al.** [43]
**Patients, n**	1	46	4
**G2EC, n (%)**	1 (100)	8 (17.4)	4 (8.3)
**Nulliparous, n (%)**	1 (100)	25 (54.3)	n.d.
**Primiparous, n (%)**	0 (0)	21 (45.7)	n.d.
**History of infertility (%)**	0 (0)	n.d.	n.d.
**Attempted to conceive, n (%)**	0 (0)	5 (10.9)	0 (0)
**Time to conception attempt after CR (months)**	-	n.d.	-
**ART (%)**	-	n.d.	-
**Pregnancies, n**	0	1	0
**Miscarriages, n (%)**	-	0 (0)	-
**Ectopic pregnancies, n (%)**	-	0 (0)	-
**Twin pregnancy, n (%)**	-	0 (0)	-
**Preterm delivery, n (%)**	-	0 (0)	-
**Full-term delivery, n (%)**	-	1 (100)	-
**Live births (%)**	-	1 (100)	-
**PR (%)**	-	20	-
**MR (%)**	-	0	-
**LBR (%)**	-	100	-

(**a**) G2EC—grade 2 endometrial cancer; BMI—body mass index; IUD—intrauterine device; LNG-IUD—levonorgestrel-releasing intrauterine device; CR—complete response; PR—partial response; SD—stable disease; NED—no evidence of disease. * For these studies, disaggregated data were not available. (**b**) CR—complete response; ART—assisted reproductive technology; PR—pregnancy rate; MR—miscarriage rate; LBR—live birth rate. * For these studies, disaggregated data were not available.

**Table 5:** (**a**) Oncological outcomes of studies reporting the use of a combined approach for assessing the FST in IA G2 ECs. (**b**) Reproductive outcomes of studies reporting the use of a combined approach for assessing the FST in IA G2 ECs.

**(a)**									
	**Chae et al.** [23] *	**Falcone et al.** [30]	**He et al.** [32]	**Hwang et al.** [33]	**Kim et al.** [36]	**Laurelli et al.** [38]	**Lee et al.** [39] *	**Newtson et al.** [40]	**Yu et al.** [47]
**Patients, n**	118	23	25	5	1	21	54	1	8
**G2EC, n (%)**	11 (9.3)	23 (100)	3 (12)	5 (100)	1 (100)	1 (4.8)	44 (81.5)	1 (100)	8 (100)
**Age (years, mean or median)**	37 (28–45)	34.6 ± 4.73	34.13 ± 4.73	30.4 ± 5.3	13	39	34 (18–44)	25	27.6 ± 4.4
**BMI, kg/m^2^ (mean)**	22.7 (18.5–43.5)	28.3 ± 5.9	26.35 ± 5.59	24.0 ± 4.5	24.8	24.3	24 (16–40)	37	30 ± 4.1
**Combined treatment regimen (% of patients treated)**	MPA 500–1000 mg + LNG-IUD	HR + LNG-IUD (52.2) HR + oPT (21.7) LNG-IUD only (17.4) oPT only (4.3) LNG-IUD + oPT (4.3)	MPA 250 mg (66.7) MPA 250 mg + GnRHa (33.3)	MPA 500 mg + LNG-IUD	MA 160 mg + MPA 10 mg + LNG-IUD (100)	HR + LNG-IUD	oPT with LNG-IUD (57.4)	MA 80 mg bid + LNG-IUD (100)	Patient 2: MA 160 + GnRHa + LNG IUD (12.5) Patient 5: GnRHa + LNG-IUD + aromatase inhibitor (12.5)
**Treatment duration (months, mean or median)**	12.2 (3–49)	17.3 ± 16.9	5 (3–18)	11.0 ± 6.2	8	6	11 (3–30)	10	4.7 ± 2.2
**CR, n (%)**	71 (60.2)	18 (78.2)	3 (100)	3 (60)	1 (100)	0 (0)	39/54 (72.2)	1 (100)	7 (87.5)
**Time to** **CR (months, mean or median)**	6 (3–33)	13 (6–77)	14.7 ± 10.1	11.0 ± 6.2	8	-	10 (3–24)	10	4.7 ± 2.2
**PR or SD, n (%)**	47 (39.8)	5 (21.7)	0 (0)	2 (40)	0 (0)	1 (100)	27.8	0 (0)	1 (12.5)
**No. of recurrence (%)**	18 (15.3)	7 (41.2)	0 (0)	1 (20)	0 (0)	0 (0)	15 (38.5)	0 (0)	3 (37.5)
**Time to recurrence (months)**	15.0 (4–48)	32.8 ± 42.3	-	23	-	-	23 (3–101)	-	25.7 ± 7.9
**Recurrence diagnosis (%)**	G1EC (83.3) G2EC (16.7)	G1EC (16.7) G2EC (83.3)	-	G2EC (100)	-	-	G1EC (80) G2EC (20)	-	AH (66.6) G2-3EC (33.3)
**Retreatment, n (%)**	14 (77.8)	1 (14.3)	-	MPA 500 mg + LNG-IUD (100)	-	-	9/15 (60)	-	MA 160 mg bid (66.6) GnRHa + LNG-IUD (33.3)
**Response to retreatment, n, % (months)**	12/18, 66.7 (15 ± 17.5)	1/1, 100 (6)	-	1 (100)	-	-	n.d.	-	2/3, 66.6 (4.3 ± 1.2)
**Hysterectomy (%)**	6 (33.3)	6 (85.7)	0 (0)	1 (20)	0 (0)	1 (100)	11/54 (20.4)	0 (0)	3/8 (37.5)
**Follow-up** **(n,%)**	NED (12, 66.6) AWD (6, 33.3)	NED (22, 95.7) AWD (1, 4.3)	NED (3, 100)	NED (5, 100)	NED (100)	NED	NED (45/54, 83.3) AWD (14.8) Death (1.9)	NED	NED (8/8, 100)
**Follow-up time (months)**	19.7 ± 18.1	59.6 ± 45.1	97.3 ± 2.1	42.4 ± 23.4	n.d.	76	44 (1–132)	15	36.2 ± 16.1
**(b)**									
	**Chae et al.** [23] *	**Falcone et al.** [30]	**He et al.** [32] *	**Hwang et al.** [33]	**Kim et al.** [36]	**Laurelli et al.** [38]	**Lee et al.** [39] *	**Newtson et al.** [40]	**Yu et al.** [47]
**Patients (n)**	49	23	21	5	1	1	54	1	8
**G2EC, n (%)**	11 (9.3)	23 (100)	3 (12)	5 (100)	1 (100)	1 (4.8)	44 (81.5)	1 (100)	8 (100)
**Nulliparous, n (%)**	44 (89.8)	19 (82.6)	n.d.	5 (100)	1 (100)	1 (100)	54/54 (100)	1 (100)	n.d.
**Primiparous, n (%)**	5 (10.2)	4 (17.4)	n.d.	0 (0)	0	0	0	0	n.d.
**History of infertility, n (%)**	n.d.	0 (0)	n.d.	n.d.	0 (0)	0 (0)	11/54 (20.4)	0 (0)	3 (37.5)
**Attempted to conceive, n (%)**	49 (41.5)	10 (43.5)	12 (57.1)	2 (40)	0 (0)	0 (0)	15/54 (38.5)	0 (0)	3 (37.5)
**Time to conception attempt after CR (months)**	7.67 (0–44)	n.d.	n.d.	n.d.	-	-	n.d.	-	n.d.
**ART, n (%)**	27 (90)	2 (20)	5 (83.3)	2 (100)	-	-	n.d.	-	n.d.
**Pregnancies, n**	30	5	6	1	0	0	7	0	2
**Miscarriages, n (%)**	7 (23.3)	2 (40)	0 (0)	1 (50)	0 (0)	0 (0)	2	0 (0)	0 (0)
**Ectopic pregnancies, n (%)**	1 (3.3)	0 (0)	0 (0)	0 (0)	0 (0)	0 (0)	0	0 (0)	0 (0)
**Twin pregnancy, n (%)**	2 (6.7)	0 (0)	0 (0)	0 (0)	0 (0)	0 (0)	0	0 (0)	0 (0)
**Preterm delivery, n (%)**	1 (3.3)	2 (40)	0 (0)	0 (0)	0 (0)	0 (0)	0	0 (0)	0 (0)
**Full-term delivery, n (%)**	20 (66.6)	1 (20)	6 (100)	0 (0)	0 (0)	0 (0)	7	0 (0)	2 (100)
**Live births, n (%)**	21 (70)	3 (60)	6 (100)	0 (0)	0 (0)	0 (0)	5	0 (0)	2 (100)
**PR (%)**	61.2	50	57.1	50	-	-	46.7	-	66.6
**MR (%)**	23.3	40	0 (0)	100	-	-	28.6	-	0
**LBR (%)**	42.9	30	42.9	0	-	-	54	-	100

(**a**) AH—atypical hyperplasia; G1EC—grade 1 endometrial cancer; G2EC—grade 2 endometrial cancer; G3EC—grade 3 endometrial cancer; BMI—body mass index; oPT—oral progestin therapy; MA—megestrol acetate; MPA—medroxyprogesterone acetate; GnRHa—gonadotropin releasing-hormone antagonist; LNG-IUD—levonorgestrel-releasing intrauterine device; HR—hysteroscopic resection; CR—complete response; PR—partial response; SD—stable disease; NED—no evidence of disease; AWD—alive with disease. * For these studies, disaggregated data were not available. (**b**) CR—complete response; ART—assisted reproductive technology; PR—pregnancy rate; MR—miscarriage rate; LBR—live birth rate. * For these studies, disaggregated data were not available.

## Data Availability

Not applicable.

## Supplementary Materials

The following supporting information can be downloaded from https://www.mdpi.com/article/10.3390/biom14030306/s1, Table S1: Modified Newcastle–Ottawa scale items; Table S2: Risk of bias assessment.
