# Supplementary material for: Reproductive and Oncologic Outcomes in Young Women with Stage IA and Grade 2 Endometrial Carcinoma Undergoing Fertility-Sparing Treatment: A Systematic Review
Source: Biomolecules. 2024 Mar 5;14(3):306. doi: 10.3390/biom14030306 (PMC10968417; doi:10.3390/biom14030306)
Supplement: Supplementary file 1 [file biomolecules-14-00306-s001.zip › Table S2.pdf]

Author	Study design and sample representativeness	Sampling technique	Description of the fertility-sparing treatment	Quality of population description	Incomplete outcome data	Total score	Risk of bias
Andress et al. [28]	-	-	★	★	★	★★★	Low
Brown et al. [29]	-	-	★	★	★	★★★	Low
Chae et al. [23]	-	-	★	★	★	★★★	Low
Falcone et al. [30]	-	-	★	★	★	★★★★	Low
Gotlieb et al. [31]	-	-	★	-	★	★★	High
He et al. [32]	-	-	★	★	★	★★★★	Low
Hwang et al. [33]	-	-	★	★	★	★★★	Low
Imai et al. [34]	-	-	★	-	★	★★	High
Kaku et al. [35]	-	-	★	★	★	★★★	Low
Kim et al. [36]	-	-	★	★	★	★★★	Low
Koskas et al. [37]	-	-	-	★	-	★	High
Laurelli et al. [38]	-	-	★	★	★	★★★	Low
Lee et al. [39]	-	-	★	-	★	★★★	High
Newtson et al. [40]	-	-	★	★	★	★★★	Low
Pal et al. [41]	-	-	★	★	-	★★	High
Park et al. [42]	-	-	★	-	★	★★★	High
Roberti Maggiore et al. [43]	-	-	★	★	★	★★★	Low
Rossetti et al. [44]	-	-	★	★	-	★★	High
Sardi et al. [45]	-	-	-	-	★	★	High
Shan et al. [46]	-	-	★	★	★	★★★	Low
Yu et al. [47]	-	-	★	★	★	★★★	Low
Zuckerman et al. [48]	-	-	★	-	★	★★	High

Table S2. Risk of bias assessment.
